# Risk evaluation at municipality level of a COVID-19 outbreak incorporating relevant geographic data: the study case of Galicia

**DOI:** 10.1038/s41598-021-00342-2

**Published:** 2021-10-28

**Authors:** Alejandro Carballosa, José Balsa-Barreiro, Adrián Garea, David García-Selfa, Ángel Miramontes, Alberto P. Muñuzuri

**Affiliations:** 1 Group of Nonlinear Physics, Faculty of Physics, University of Santiago de Compostela, 15782 Santiago de Compostela, Spain; 2 Group of Territorial Analysis, Institute IDEGA, University of Santiago de Compostela, Santiago de Compostela, Spain; 3 MIT Media Lab, Massachusetts Institute of Technology, 75 Amherst St, Cambridge, MA 02139 USA; 4 CESGA (Supercomputing Center of Galicia), Avda. de Vigo s/n, Campus Sur, 15705 Santiago de Compostela, Spain

**Keywords:** Nonlinear phenomena, Dynamical systems, Dynamic networks

## Abstract

The COVID-19 pandemic was an inevitable outcome of a globalized world in which a highly infective disease is able to reach every country in a matter of weeks. While lockdowns and strong mobility restrictions have proven to be efficient to contain the exponential transmission of the virus, its pervasiveness has made it impossible for economies to maintain this kind of measures in time. Understanding precisely how the spread of the virus occurs from a territorial perspective is crucial not only to prevent further infections but also to help with policy design regarding human mobility. From the large spatial differences in the behavior of the virus spread we can unveil which areas have been more vulnerable to it and why, and with this information try to assess the risk that each community has to suffer a future outbreak of infection. In this work we have analyzed the geographical distribution of the cumulative incidence during the first wave of the pandemic in the region of Galicia (north western part of Spain), and developed a mathematical approach that assigns a risk factor for each of the different municipalities that compose the region. This risk factor is independent of the actual evolution of the pandemic and incorporates geographic and demographic information. The comparison with empirical information from the first pandemic wave demonstrates the validity of the method. Our results can potentially be used to design appropriate preventive policies that help to contain the virus.

## Introduction

As of Nov 15th, 2020, the SARS-Cov-2 or COVID-19^1,2^ has left in Galicia, an autonomous community in Spain, a total of 43,714 cases, 5783 hospitalized, 661 in ICU and 1084 deaths^3^. Comparatively, these represent 2.9% of the total cases and 2.6% of the deceased nationwide. The ratio of infected to 100,000 inhabitants is 161.9 and the death ratio is 4 per 100,000 inhabitants. The fatality rate is 2.5 deaths for every 100 confirmed cases and 40.1 deaths for every 100,000 inhabitants. The first of these two ratios is in accordance with the rest of Spain and the second in the order of half (compared to Spain). The incidence of the so-called *first wave*, as of June 30th, showed a total of 10,853 cases and 619 deaths in this region. The fatality rate during this first wave was 5.9 deaths per 100 confirmed cases and 22.3 deaths per 100,000 inhabitants. The seroprevalence study carried out by the Galician government during the first wave estimates that only 1.15% of its population had passed the virus at that time^4^. Most recent studies estimate that this rate would reach 4.5% in December 2020, that means, lower than half than for the whole Spain^5^. Therefore, Galicia was one of the least affected regions in Spain.

By means of differential equations and a few tools of complex networks, we have simulated the geographical spread of the virus among the different municipalities (third-level national administration entities) that make up the region (Galicia). Some authors have already presented their concerns on the long term predictability of epidemiological models when describing large real-world systems and have compared it to the process of weather forecasting, where knowing the precise values of several variables only allows for short-term forecasting in matter of a few days^6–8^. Accurately modelling all the small details that concerns the spreading of a virus, from biological features to social interaction, is a hugely difficult task that one model alone cannot comprehend and thus model predictions should be treated carefully. One important point to address is that the mobility of people across the country results in the sudden and unpredictable emergence of virus outbreaks and so understanding the mobility patterns of human population has been the focus of many previous studies in order to better understand the virus spreading process^9–14^. Another remark to consider is that each population is geographically unique, has its own history, economic resources, and demographic circumstances. Unveiling which communities have a potentiality of becoming hotspots of the epidemic and which ones do not is crucial for understanding the large-scale behavior of the virus spread and therefore for policy making regarding any kind of mobility restrictions^15,16^ as well as previsions in terms of medical reinforcements. Based on this, the different municipalities that conform the Galician territory are considered as nodes of human population in the abstraction of our mathematical model. Links connecting these nodes shape the structure of a network and act as the different means of transportation between the localities, each one possessing a real number or “weight” that accounts for the flux of pendulum movements of commuting among the nodes. The larger the population density or the shorter the time distance between nodes, the stronger will be the weight of the link connecting them. Using this approach, we are able to differentiate between the most populated areas that act as hubs for human and economic activity, their surrounding localities that usually serve as dormitory towns and the rural areas, less populated and quiet. Thus, one should think of this set of nodes and links as the skeleton of the model accounting for the spatial distribution of population across the territory, while more layers of complexity are required for a more precise approximation to the real picture.

The use of complex networks for accommodating infective transmission dynamics is an extensive field of research within the complex systems science, being a topic covered in several books and reviews^17–20^. A usual approach here is to consider each node of the network as a single individual and the edges between them as face-to-face interactions that occur daily and model the infective transmission of the disease as a stochastic process that occurs with a certain probability through the edges of the network. This scheme that traces the mobility of the individuals^21^, allows for simulations of entire populations that scale down to the individual interactions with their closest circles and local neighborhoods, being these interactions usually modelled by adding geographical and demographical data to the nodes and simulating the daily interaction patterns^22^. With this information at hand, one can effectively simulate the outcome of vaccination schemes targeting specific nodes or group of nodes^23,24^, or analyze how non-pharmaceutical interventions such as social distancing or mobility restrictions can hinder the spread of the viral disease^25,26^. Note that these strategies that aim to restrain the diffusion of the epidemic dynamics usually imply altering the connectivity pattern of the network, either by blocking nodes that act as hubs or super spreaders within the network or by cutting down the number of interactions and/or edges between the nodes. A comparison between the effectiveness of these methods and others has been carried out by^27^.

A different scheme considers nodes as entities where multiple individuals can be located, acting as subgroups or subpopulations of a larger whole. Note that in this way one can attribute certain geographical characteristics to the nodes to naturally include the spatial distribution of a set of localities within a territory. This approach is known as *metapopulation network models*^10,28,29^ and here the infective transmission process occurs locally, where one could think of individuals as particles interacting with each other, while these are allowed to simultaneously travel through the edges of the network according to a set of rules and diffusing the disease through multiple nodes. These network models also allow the possibility of considering a deterministic nature to the infection process instead of the stochastic one, exploiting the features of the classic family of SIR-like models^30^ with an added spatial dimension^16^. The SIR model distributes the population into three exclusive compartments: *Susceptible*, *Infected* and *Recovered*. Individuals from the first two groups are allowed to interact increasing the net amount of infective cases in time. Note, however, that these models assume that the whole population is homogeneously distributed in space, an assumption that can be overcome with the inclusion of the metapopulation network. With this consideration in mind, one can simulate the dynamics of the epidemics in a large heterogeneous population based on a computationally affordable way.

After reviewing the different methods and as we previously advanced, here we opted for the latter approach. It is known that deterministic epidemiological models have proven to be quite insightful in its simplest forms^31–33^, while more elaborate variants have been successfully implemented in tackling the virulent spread of the current coronavirus disease 2019 (COVID-19) in the cases of the city of Wuhan^34,35^, China^36^, Italy^37^ or Portugal^38^, among others. Metapopulation network models with deterministic nature have also been applied to the dynamic modelling of COVID-19^15,16^ in order to better understand and assess the nation-wide spread of the virus and lockdown relaxation measures. Motivated by these works, we have adapted the model developed in^38^ to the Galician network. It consists in a variation of the classic SIR model, with the inclusion of one compartment for Exposed E or yet asymptomatic individuals, and another compartment for Protected P individuals, introducing in the system the presence of non-pharmaceutical interventions. Rather than providing an optimal fit to gathered incidence data on active cases, our goals are closer to those of^16^, with the aim pointed towards the large-scale picture and finding which nodes are more likely to suffer an outbreak of the pandemic just by its situation on the network and socioeconomic reality, i.e. the weak spots in the network. In contrast with the cited works, where the adjacency matrix of the network was modelled based on actual commuting census data, our study addresses a suitable approach to reconstruct the locality interactions in the absence of such databases, incorporating further demographic data of interest such as age structure or harbor activity (relevant for Galicia). Given the outcomes of the simulations and a given set of considerations, we are able to associate what we shall name a risk factor to each one of the nodes.

As expected from the observed patterns of the virus spread^16^, the capital cities headed the rank while at the same time induced a strong influence on its closest neighboring nodes, especially in the case of the two most populated cities, Vigo and A Coruña. Enhancing the exposure to the virus on nodes with higher economic and human activity known beforehand helped to noticeably increase the risk factor on less populated nodes, while rural aged nodes with lesser human activity resulted on a small-to-none risk of suffering an outbreak. While the calibration of the model was carried out following^38^ with data at a regional level, a comparison of the results at node level with official data of cumulative cases showed a high degree of correlation.

An independent mathematical analysis based only on the linear stability of the model dynamics and the given network was also carried out, reassuring the performance of the model. This type of analysis based on the termed linear growth rates or growth factors, has been applied before in order to inspect if particular nodes of a complex network are more unstable than others, giving rise to node differentiation and predicting the emergence of mathematical abstractions such as the Turing instability^39–41^. Extrapolating the approach to the infection transmissive dynamics, the information underlying in the network sufficed to achieve similar results to those of the detailed simulation based on numerical integration of the model.

Finally, notice that although the Galician territory has been considered as a study case, the developed approach to assign risk factors to each locality could be extrapolated to other regions, work which we intend to do in the future.

### Study area

Our study area is centered in the region of Galicia, the northwest corner of Spain. It counts with 2.7 million inhabitants distributed in almost 30,000 km^2^, showing a population density of 91.3 people per square Kilometer^42^. In administrative terms, this region is divided into four provinces (second-level territorial and administration units), three in contact with the coast and one inland. Galicia counts with 313 municipalities (third-level territorial and administration units), which correspond to local government units. The network of nodes in which we work in this paper is extracted from these territorial units counting with 313 nodes in total.

**Figure 1:**
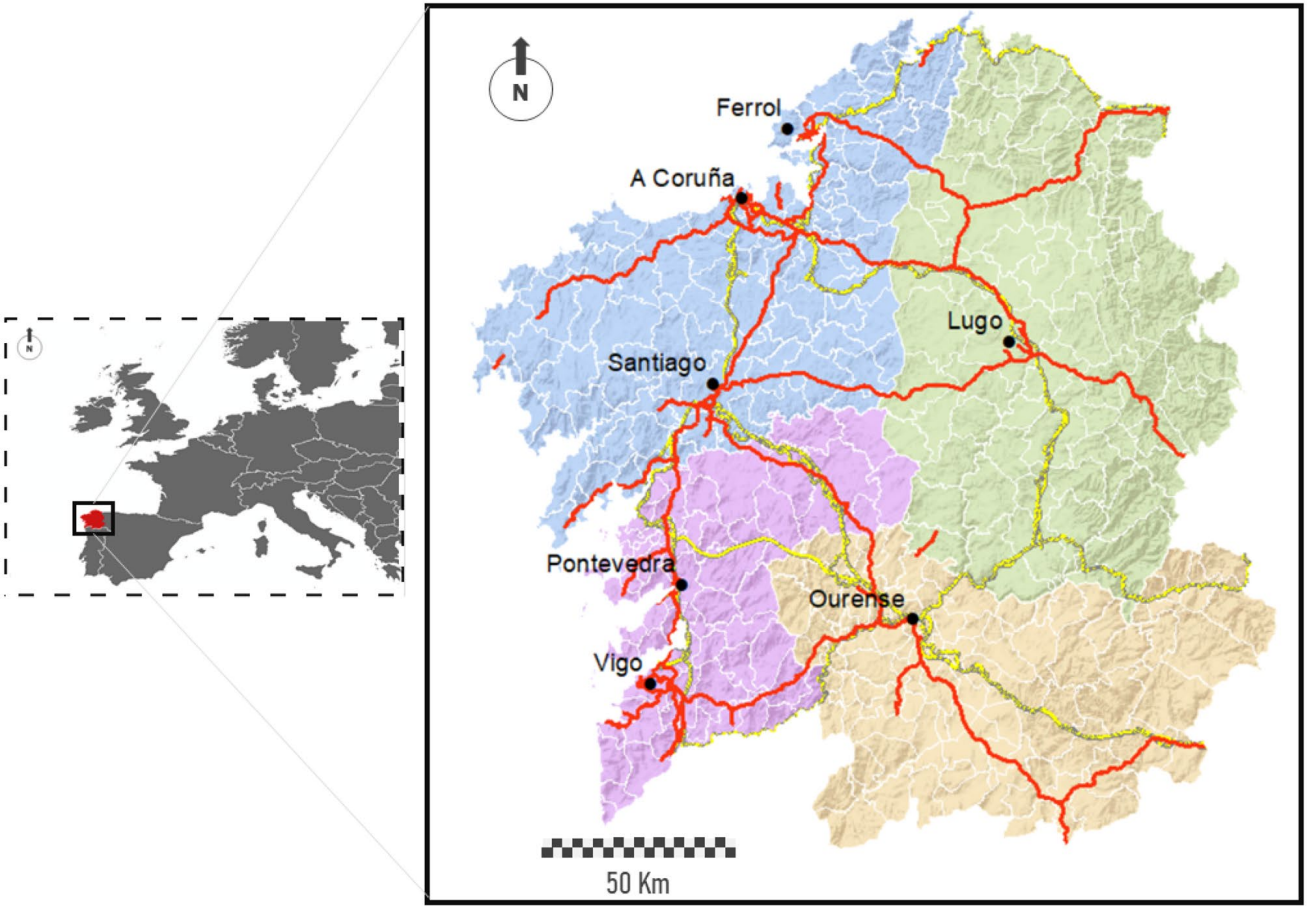
Location and characterization of the Galician region. In colored background, distribution in provinces (second-level administrations) and municipalities (third-level administrations). The most populated cities and industrial hubs of each province appear highlighted in bold. Red lines represent the major road infrastructures, i.e., motorways in red and railways in yellow. Maps are generated using ArcGIS Desktop software version 10.8 (from ESRI, https://www.esri.com/en-us/arcgis/products/arcgis-desktop/overview).

Traditionally, its spatial pattern of population distribution is characterized by high dissemination across the territory^43^. In fact, this region concentrates almost 50% of all the singular population entities in Spain^44^. This rate is about 10 times larger than its demographic weight at a national level. In the last decades, a decline of this traditional pattern of population distribution has been observed, being evident a tendency to the concentration of population. Currently, most of the population concentrates close to the so-called Atlantic Axis, the area surrounding the AP-9 motorway that links northern Galicia with Portugal. This area is located in the west side of the region, very close to the coastline. The major and most thriving cities in this region, five of the seven most populated ones, are located along this axis. Vigo and A Coruña are the two most important cities with approximately 300,000 inhabitants each. The rest of the major cities have populations of about 100,000, with the exception of Pontevedra and Ferrol, which are less populated. In Fig. 1 the seven major cities are also shown, namely, A Coruña, Ferrol, Lugo, Ourense, Pontevedra, Santiago and Vigo. From an urban perspective, this region is characterized by the lack of intermediate cities (between 30,000 and 100,000 people). In the last decades, some municipalities surrounding the metropolitan area of the most important cities (such as Narón, Arteixo, Ames, etc.) experienced the emergence of a growing group of intermediate cities^45^.

The urban pattern of this region shows a hierarchy dominated by the western sector, which presents in the surroundings of the Atlantic Axis, positive demographic dynamics both from a quantitative (higher fertility rates, positive migratory balances) and qualitative perspective (less aged population and more dynamic demographic structures). Just the opposite occurs in the eastern sector where the two main urban references are the two province’s capitals, which concentrate a large part of the socioeconomic activity of their provinces. The rest of the territory, which is more rural in nature, is articulated based on a group of head towns that dominate large spaces, whose influence goes beyond their administrative units.

Another important socio-economic factor in Galicia is its close relation with the sea. In fact, along its more than 1700 km of coastline, it has 128 ports. 122 ports are the responsibility of the regional government and 6 are managed by the Spanish government. The sea related activities in these ports is multiple and involves aspects ranging from purely fishing activities (deep-sea fishing activities, inshore, shellfish, commercial activities with loading and unloading of all kinds of merchandise), passengers (regular transport and cruises), recreational and tourist nautical activities. This generates a series of social and economic consequences with great significance for Galicia and, therefore, with important consequences within this pandemic context as we will explain bellow^46,47^.

It is also noteworthy the age distribution in the region, in fact the life expectancy is one of the largest in the world (83.3 years in 2020) and the total population above retiring age is significantly large (25.5% in 2020). This is the second more aged region, with an *aging index* of 207.3% (rate estimated based on the number of people older than 64 years related to 100 people younger than 16 years), a value much higher than the national average (129.2%)^42^. Another important related factor is linked to the traditional structure of family units where most of the elders remain in their homes or in close-relatives homes. In fact, the rate of beds in nursing homes per 100 elderly people is significantly lower than in the rest of Spain^48^. Thus, in order to keep simpler our model, we discard the effect of nursing homes in the evolution of the pandemic and instead increase the protection of aged municipalities.

## Results

In this section, we present the territorial impact of the COVID-19 in the Galician region, a plausible model that simulated the infection process and two equivalent methods to estimate a local outburst risk factor. In order to fit the parameters in our model we considered the cumulative incidence data officially gathered for the Galician region during the so-called first wave of the pandemic, this was, from March 1st to around June 30th, 2020. Note that this period of time corresponds with the first pandemic wave in Spain, the one that was less influenced by governmental interventions.

### Territorial impact of COVID-19 disease

We considered data in this region from the start of the epidemic until June 30th, 2020. The Galician Health Service (SERGAS), the competent authority in health management in the region, provided this data^3^. In short, the database counts with 10,853 cases with their corresponding locations. The location of these cases allows us to evaluate the spatial distribution of the disease and to understand its spatial dynamic. Data are aggregated in municipalities and these are displayed in choropleth maps.

**Figure 2:**
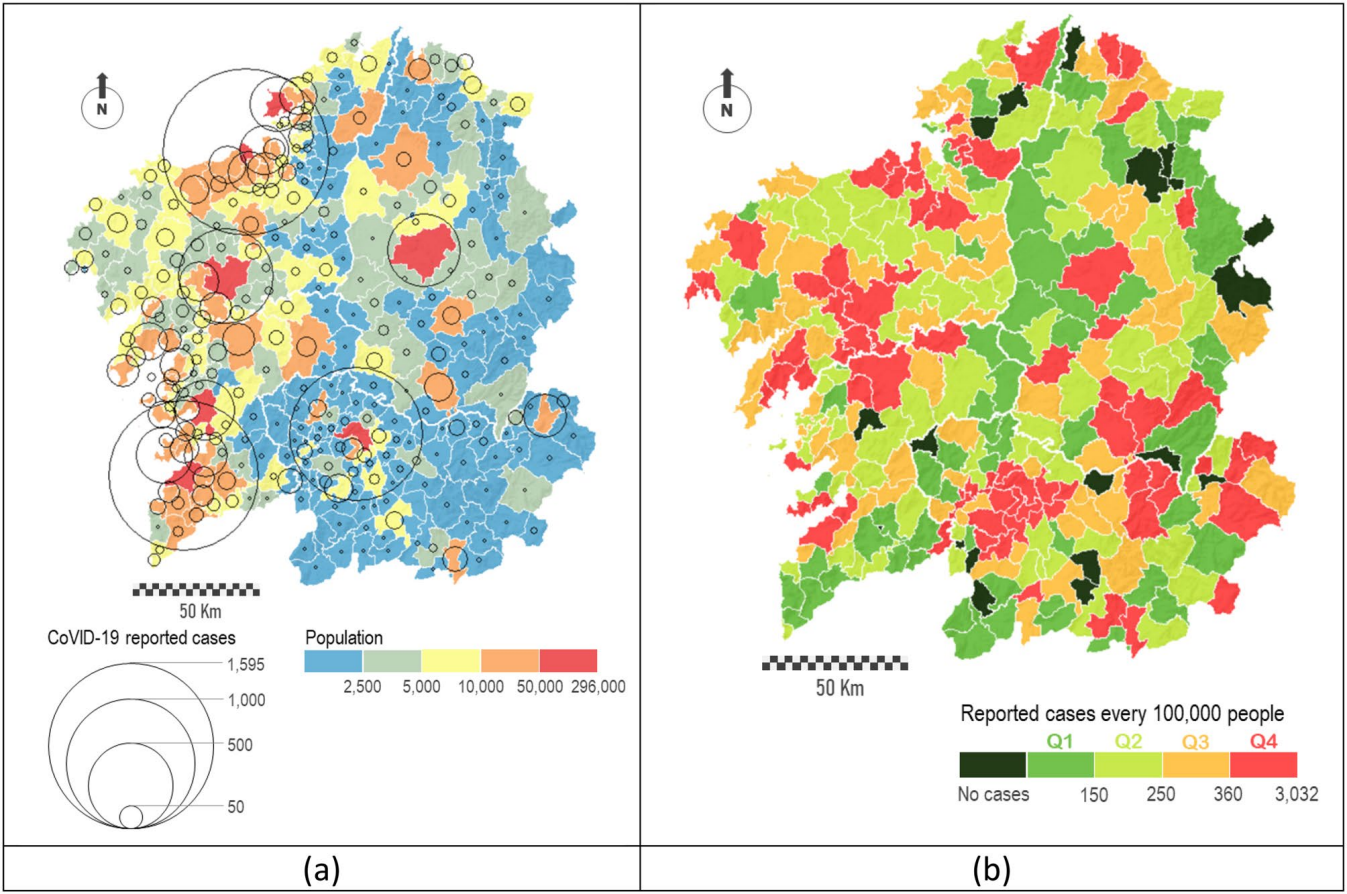
Geographical spread of COVID-19 in Galicia at a municipal level: (**a**) Reported cases versus total population, and (**b**) Incidence rate. Maps are generated using ArcGIS Desktop software version 10.8 (from ESRI, https://www.esri.com/en-us/arcgis/products/arcgis-desktop/overview).

On the one hand, the geographical spread of the virus was clearly more significant in the western sector of the Atlantic Axis, especially in the areas close to the major cities. Figure 2a relates the number of reported cases with the total population. Reported cases are represented by proportional circles, whereas the total population is distributed in five defined intervals, ranging from purely rural to urban municipalities. A priori, it is observed a strong spatial correlation between the total population and the number of reported cases in each municipality. A more exhaustive analysis from a geographical perspective is shown in Fig. 2b, where we represent the incidence rate of the disease, i.e., the number of cases every 100,000 people. Here, we observe how the peri-urban areas surrounding the major cities acquire more prominence. It is explained because in these areas is where most of the people live (residential areas), but also where the industrial activities are mostly concentrated. Therefore, these spaces present a higher dynamism based on a higher number of social interactions.

### Parameter estimation and model calibration

The mathematical equations describing the propagation of the epidemic are described in detail in the Methods section and in ref.^38^. This model considers five distinct groups of population and the interaction among them and has been previously used to successfully describe the evolution of the pandemic elsewhere. Several parameters describe the interaction between the population categories and characterize the transitions from one category into another, being the most relevant the infective transmissive rate $\beta$, the fraction of susceptible individuals that enter and exit the protected compartment, $p$ and $m$, and the parameter $d$ that modulates the coupling between nodes of the network. This model considers several layers of information relevant to the study case (see “Methods” section for details), in particular, information of the net flow of passengers coming through the airports was included. Another layer includes an additional term in the infective transmissive rate, $\beta$, due to the port activity that it is so important in the coastal area of Galicia. The last layer of information includes an indication of the economic activity (measured via the average age of each location) as more economically active nodes are more prone to interact with others and, thus, have a negative impact in the spread of the disease.

**Figure 3:**
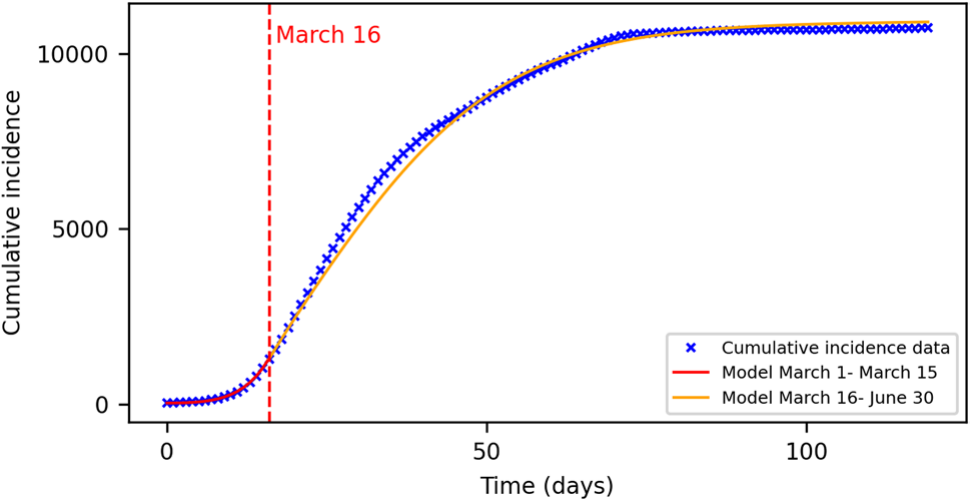
Estimated cumulative incidence compared to the real data. The fitting process of the model was separated on the two shown time-regions, one before the start of the lockdown and one after. The initial condition for the simulation was extracted from the first measured value of the dataset, and the number of cumulative cases distributed proportionally among the four capital cities of the provinces and the capital of the region (Santiago de Compostela).

The parameter estimation is extracted from the mathematical model by superposition of the infected and recovered/removed compartment at each time $t$ of the simulation, stablishing a straight-forward link between data and model and allowing us to fit the differential equations by means of a non-linear least squares minimization protocol^38^. Since the available data is at regional level, the fitting process was performed considering all mentioned layers of complexity (see “Methods” section for details) and the sum of the compartments across all nodes in the network. I.e., the model was fitted to the big picture and not at the local level (single node level). Detailed data at the local level is usually hard to find and, in most of the cases, it is endowed with a larger error. Another important remark about the experimental data is that the imposition of a lockdown and subsequent restriction measures resulted in an irregular dataset that can only be well parametrized by time-variations of the transmissive infection rate, as already was pointed out by refs.^15,16^. In the case at hand, the strong measures imposed by the national government set down a clear turning point on which the daily incidence of the virus started to decelerate and to slowly decrease in time. With this in mind, we have considered two different time regions with its own set of model parameters, separated by the time when the lockdown started. Specifically, during the fitting process we left free three of the model parameters, the infective transmission rate $\beta$, the network coupling $d$ and the fraction of people that leave protective measures $m$. The time duration of the second region goes until the isolation measures were strongly relaxated and a state of “new normality” emerged during the summer 2020. The estimated curve of cumulative incidence is shown on Fig. 3 for both temporal situations, and the parameter values are presented on Table 1. The initially infected individuals were distributed along the regional capital cities for simplicity (30 initially infected individuals distributed along the major cities proportionally to their local population).

**Table 1:** Summary of the parameter values used in the simulations presented along the text.

Parameters	1st period (Mar. 1st–Mar. 16th, 2020)	2nd period (Mar. 16th–Jun. 30th, 2020)	References
$\beta$	$1.0692$	$0.3735$	Estimated
$m$	$0.2882$	$0.01$	Estimated
$d$	$1e - 6$	$1e - 6$	Estimated
$p$	Age dependent, see “Methods” section		
$\phi$	$1/15 d^{ - 1}$		Time until lockdown
$w$	$1/75 d^{ - 1}$		Time until lifting the lockdown
$\mu$	$1/30 d^{ - 1}$		^38^
$\nu$	$0.15$		^38^

**Figure 4:**
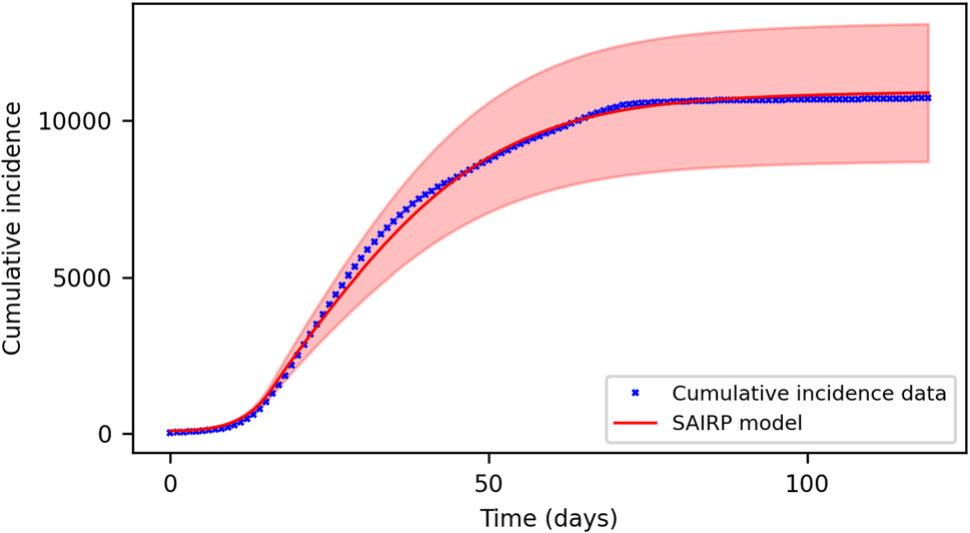
SAIRP network model with different random realizations of the initial state. The red line represents the mean value of the cumulative incidence over the whole number of carried simulations, while the red shadow represents the standard deviation of the sample.

Figure 4 presents the average behavior over 1000 realizations of the same simulation with different randomly-chosen initial conditions. The red continuous line represents the mean value of the estimated cumulative infected for the whole region, while the red shadow accounts for the standard deviation over all simulations.

### Estimation of the risk factor via numerical simulations

**Figure 5:**
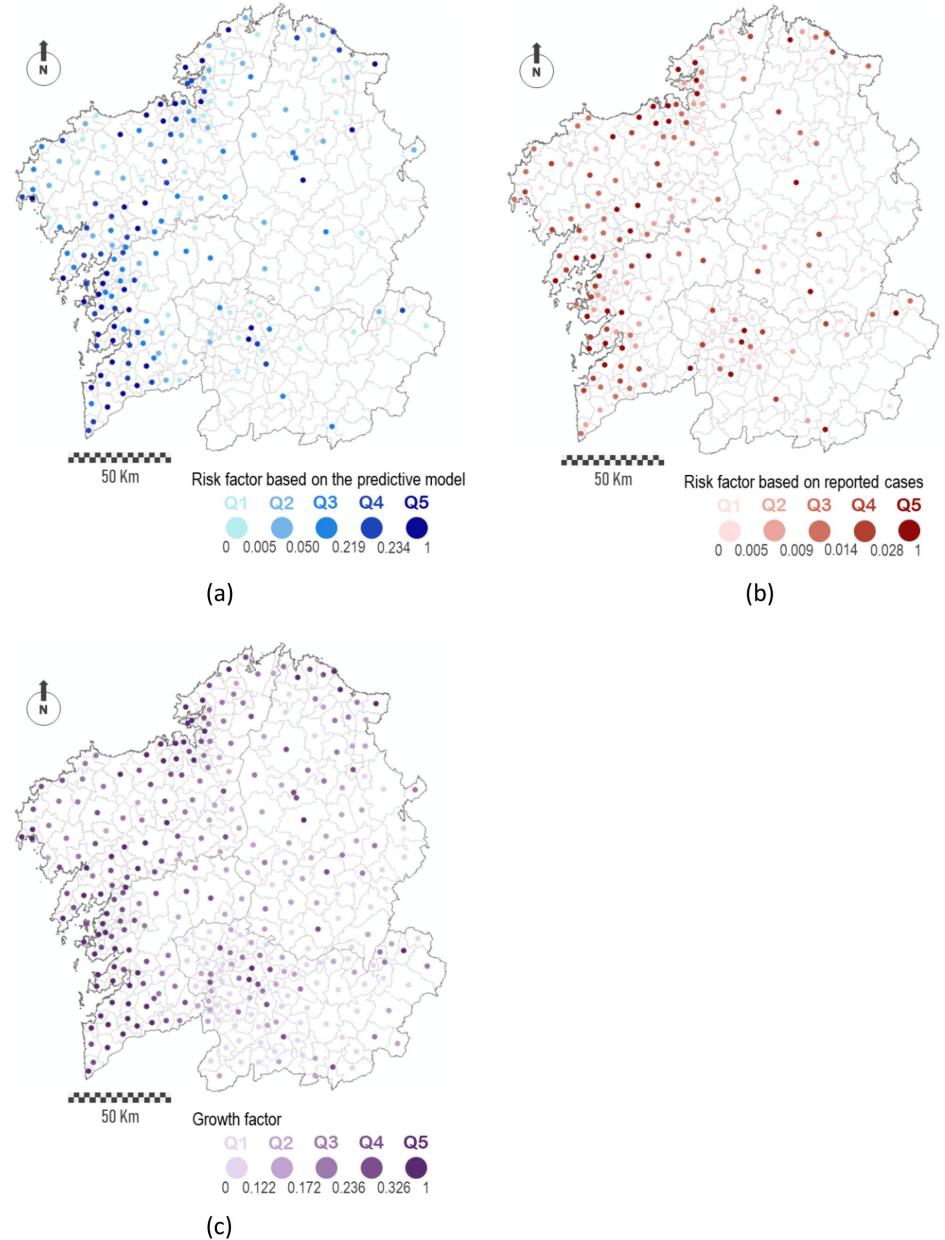
Spatial distribution of the risk factor (**a**), normalized cumulative incidence data (**b**), and growth factors (**c**) for each municipality. A value of 1 on the risk factor means that the node was found always among the top infected ones on each simulation. Following the same reasoning, a value of 0 means that that node was never found among the top ones. The ranking of the infective nodes was set with a total size of 30, and the cumulative incidence data was normalized over the highest value of reported cases. Maps are generated using ArcGIS Desktop software version 10.8 (from ESRI, https://www.esri.com/en-us/arcgis/products/arcgis-desktop/overview).

As explained in the introduction and in the Methods section, we performed multiple repetitions of the numerical simulation of the model with different randomly-chosen initial conditions (several initially infected cases randomly scattered along the different nodes in the network) in order to estimate some risk factor per node (see “Methods” section). After each simulation, we marked the locations (nodes in our network) with the larger infection peaks. Those nodes that appeared infected frequently during all the simulations were assigned with a higher value of the infection risk. On the contrary, nodes that almost never got infected during our simulations were assigned an almost null risk of infection. We have termed this value as the risk factor of the node, and we shall refer to it in this way from here on. The outcome of the risk factor estimation reproduces well the cumulative incidence of the Galician municipalities up to June 30th, as seen on Fig. 5a. Panel 5b represents the empirical number of cases per municipality (Fig. 2) on a scale from 0 to 1, normalized over the highest number of reported cases so that it can be directly compared with the risk factor used in the epidemiological model. A visual inspection of both panels demonstrates the qualitative validity of this technique.

### Estimation of the risk factor via linear stability analysis

With the parameters presented on Table 1 and considering all layers of complexity, we computed the growth factors for each node of the network (see “Methods” section) resulting in the values shown in Fig. 5c. The stability analysis is done around the steady state that it is characterized by all individuals in the susceptible compartment, this is, $\left( {\overline{{S_{i} }} ,\overline{{A_{i} }} ,\overline{{I_{i} }} ,\overline{{P_{i} }} } \right) = \left( {N,0,0,0} \right)$. This is an unstable steady state (any perturbation will trigger the epidemic). In terms of stability analysis, this implies that for the parameters considered the epidemic will outbreak given any perturbation to the initial condition, and, thus, we expect the perturbations to grow on almost all nodes with positive growth factors. A normalization with the maximum value was done for the sake of comparison with the risk factor shown in the previous section. Essentially, the stability analysis outcome is qualitatively and quantitatively equivalent to the one calculated via numerical simulations, suggesting that hubs of human activity and nodes within the *Atlantic Axis* are the most likely to suffer hotspots during an epidemic. Note that the values depicted were obtained without solving the equations but just analyzing the structure of the network combined with the type of dynamics considered per node. Quantitative differences between the growth factors and the risk factors arise due to the risk factor being computed by means of discrete summing (how many times each node appeared on the simulation rankings) while the growth factor ranges in a continue set of values.

**Figure 6:**
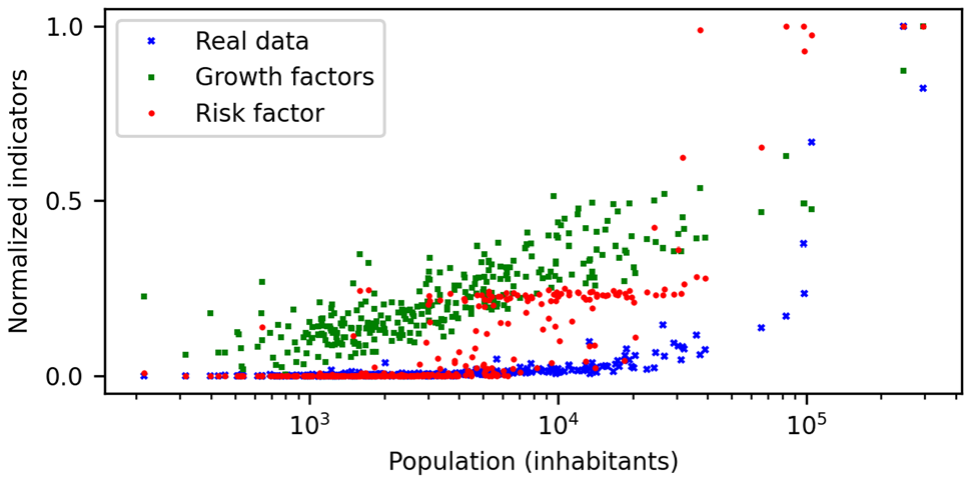
Comparative of three different normalized indicators for the municipality nodes. The figure plots the normalized cumulative incidence data (blue), risk factors (red) and growth factors (green) versus the municipality population in semi-logarithmic scale.

The distribution of the quantities analyzed against node’s population is represented in Fig. 6, as a summary of the results presented in this section. Note that the risk factors calculated via growth factors are obtained considering only structural information of the system, i.e., dynamical equations and the different layers of structural information that were incorporated (mobility, age distribution, etc.).

## Discussion and interpretation

We have modelled the geographical spread of the COVID-19 across the Galician territory during the first wave of the epidemic. With multiple random realizations of the initial seed of infected individuals, we have been able to estimate the risk factor characterizing each municipality (node in our extended network). Large values indicate a high risk to suffer an important outbreak of the virus. This risk was compared with the actual reported cases for each municipality during that time and the Spearman’s rank correlation coefficient was computed^49^. This coefficient gives us a measure of how strong the monotonic relationship between any two variables at hand is, making it ideal to compare the visible trends of our data. A positive correlation value of 0.7195 suggests that although not perfect, the model is able to capture at great extent the detailed risk for each node. An independent mathematical analysis based on the linear stability of the network dynamics was also carried out validating the previous results. The correlation value of this analysis compared to simulations is 0.9567, while the correlation with the reported data cases returned a value of 0.7421. Note that although these coefficients are merely orientative, they inform us if shared tendencies are present among the different outcomes. Keep in mind that we are actually comparing real cumulative incidence data (also endowed with some intrinsic error due to human manipulation) with the estimated risk based on our approach, not the cumulative incidence estimated with the model. Our risk assessment intends to give an orientative prediction on how likely, given similar circumstances but different initial conditions, the reported incidence would have been the same. Even more, this might become a tool for understanding the risk of a particular node and for evaluating different strategies in order to reduce it. For the most populated nodes ($\sim10^{5}$ inhabitants), we found that the risk is high in most of all possible scenarios, while for medium populated nodes things could have been completely different in more than half, either by suffering stronger outbreaks or by not experiencing the virus at all. For a majority of nodes, mostly rural and low populated (Fig. 5), the risk of an outbreak was null or practically null and this can be easily contrasted with the reported cases. It is important to note that the risks factors were evaluated through intensive simulation running multitude of experiments while the growth factors were calculated once directly from the mathematical model. The large correlation between these two factors indicates their equivalency. Thus, instead of running costly simulations, one straightforward calculation can provide the same information and help evaluate the nodes that are more likely to suffer an outburst.

Concerning the two different ways to compute the risk factor, we observe that both cases reproduce the reality up to a good extent. The main discrepancy comes from the discrete number of realizations used to compute the risk factors. The growth factors (stability analysis) grow monotonically with the population while the risk factors (direct numerical simulation) concentrate mainly in the three clusters of values observed on Fig. 6, $\sim0,\sim0.3$ and $\sim1$. It is important to remind that both methods designed to calculate the risk factors include several layers of information in order to improve the accuracy of the method. During the first pandemic wave, a complete national lockdown prevented that the Galician region would suffer from strong incidence (in comparison to the rest of Spain or even Europe) and halted drastically the spread of the virus from the capital cities to the lower populated municipalities (see Figs. 5c and 6 for a visual inspection). This was not the case for the subsequent pandemic waves in which looser restrictions were imposed and the economy was reactivated, which led to higher rates of daily incidence^50^. Table 2 presents several correlation factors between the growth factors and the experimental cumulative incidence calculated for the first and second waves and considering independently the different layers of information. The first piece of information that we observe is that all the correlation coefficients are improved in the second wave meaning that our model satisfactory predicted the risk of the different municipalities.

**Table 2:** Relevance of the different layers of information in the model for the first and second pandemic waves analyzed using different correlation parameters.

	Spearman		Pearson $r^{2}$		$R^{2}$	
Model layers	1st wave	2nd wave	1st wave	2nd wave	1st wave	2nd wave
Mobility	0.8572	0.9463	0.1283	0.1674	− 53.13	− 51.34
Ages	0.7463	0.8185	0.2332	0.3006	− 2.74	− 2.47
Multilayer	0.7421	0.8091	0.2397	0.3118	− 2.16	− 1.94

**Figure 7:**
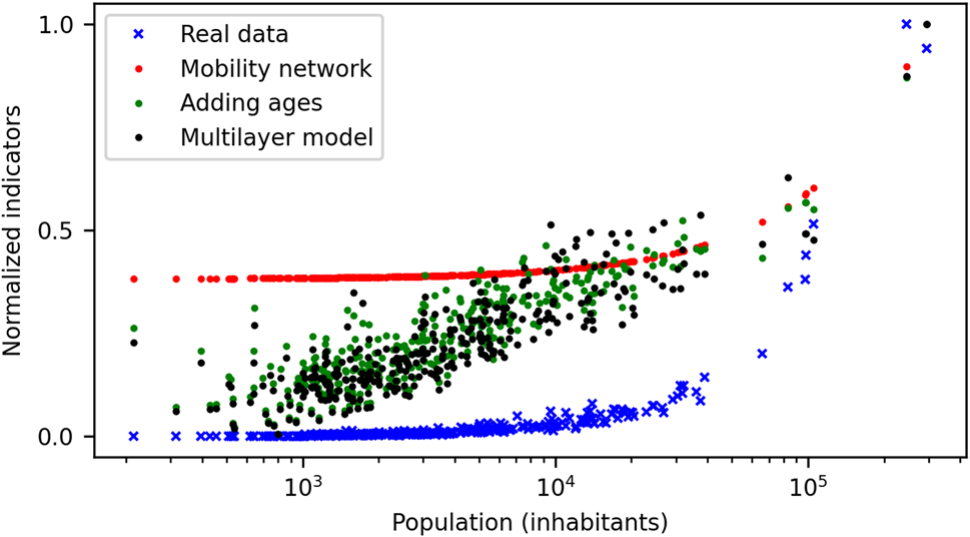
Effect of the different layers of information on the calculated growth factors. The blue crosses show the normalized cumulative incidence data of the second wave. Red dots show the growth factors calculated considering only the mobility information layer in the model. Green dots also include of the age-related economic activity and, finally, the black dots show the growth factors considering all the information layers (including port activity) described in the Method Section.

In order to understand the importance of the different layers of information we need to consider other correlation coefficients besides the Spearman’s already discussed. We have introduced the Pearson correlation coefficient for linear regression with zero intercept (values ranging from 0 to 1) and the coefficient of determination $R^{2}$ (with values from minus infinite to 1)^49^. While the Spearman returns the highest correlation when considering the model with just the mobility network, this value is biased by the fact that the Growth Factors returned for this case a very similar not-null value for almost all low-populated municipalities (see Fig. 7). Thus, the monotonous relationship between risk factors and data is almost identical and the Spearman correlation is almost one, but what the risk factors would be telling us is that low populated municipalities would have an important risk of suffering a new outbreak, which both the empirical data and the other two metrics prove wrong. On the other hand, the Pearson correlation and the R^2^ test are both based on the quantitative values of the data series and measure how much do the data series fit to a straight line. Note that the intercept of the linear fit must be fixed to zero in order to ensure that those municipalities with almost null cases also should have a very low risk factor. Otherwise, the Pearson coefficient in the mobility model would also return a misguiding correlation value.

From Table 2, we observe that increasing the layers of information results in improved correlation with the empirical data for these two metrics. Note that the low values for the Pearson coefficient and the negative ones for the $R^{2}$ are just telling us that the relation between these two series is not linear, which we already expected. We should focus mainly on the relative differences among the outcomes. When only the mobility layer is considered, the correlations are significantly worse and including the economic activity via the age distribution results in an important improvement of the model. Finally, the rest of the information considering portuary activity slightly increases the correlations. Although this small difference could be telling us that this last layer of information is an unnecessary refinement, we are firm with its importance to accurately measure the risk of the Galician portuary municipalities, at least within the framework of the methodology presented here. For different approaches in which a specific infective transmission parameter $\beta_{i}$ could be obtained directly for each municipality, e. g., fitting each subregion to its own data series, this layer of information could be discarded. The incoming of daily ships and transports to these cities and villages suppose a higher intrinsic risk of external contagions in comparison to central ones, and thus it is difficult to incorporate this information otherwise in our approach.

Focusing on territorial aspects, it is crucial not only for understanding the geographical patterns of the virus spread but also for policy making regarding the mobility restrictions of the population. We did not intend here to postulate future scenarios of lockdown neither relaxation measures with the different possible outcomes, for that we refer the reader to a study such as^15^. Our main goal was to show the difference that geographical placement and demographic circumstances has on the impact of the virus spread, a future work concerning a larger portion of territory is intended.

Comparison of both maps suggests that the mathematical model was able to accurately capture which areas were the most affected. Highly populated hubs of socioeconomic activity contain most of the nodes with higher risk of heavy infection, increasing nearest less-populated localities risk to suffer an outbreak. The so-called *Atlantic Axis*, which refers to the western territorial area connecting the two main hubs, contains almost all the highlighted nodes on the map. It is clear how the infections are primarily focused on the main cities, but also in their suburban areas. Thus, we can see how the nodes surrounding the major cities show levels of infection extreme in relative terms (Fig. 2b). Our model also correctly estimates the higher risk levels in many intermediate nodes, such in the case of the so-called *head towns*, especially those located in both western provinces. This higher infection risk is due to the increase in the number of social interactions in these towns, which concentrate a relevant socioeconomic activity such as trading centers for large areas based on primary sector. In addition, the model differentiates some areas with singular local dynamics, such as the region termed *Costa da Morte*, situated on the far north-western coast and which presents a clear regressive demographic dynamic despite being close to the most dynamic axis in this region^51^. Therefore, the implemented model is able to capture the different local dynamics present in the system.

Several aspects must be considered from a territorial approach. First, remark again that our actual data was limited to the first wave and that the analysis of the second and successive waves, ranging from July 2020 to the present date of submission is also left for future work (although some hints were already included in order to prove the robustness of the method presented). Note that this first wave of the pandemic is also the less altered by political decisions. The complexity of the virus spread may be understood when it occurs in a hierarchical way, with a concentration of cases in the most populated cities during the first weeks. It is expected that the spreading process affects lower levels of the network hierarchically (less populated nodes) over time. In addition, it should be evaluated if areas where in theory people live more concentrated (cities, although it will depend on the building density) favor a major spread of the virus more than nodes with lower risk (villages), but where community life is closer with more strong family-based relationship structures.

A second fundamental factor is the unequal territorial impact of the epidemic in terms of fatalities. This is due to some regional characteristics that act as determinants^52^. It explains how the spatial distribution of fatalities may be different to the infection maps. Initially, the virus had circulated more in the western nodes, close to the major cities, due to a larger concentration of the population and the higher socioeconomic dynamism (more interactions) in all these areas. However, the territorial impact of the pandemic in terms of fatalities is probably more important in some eastern sectors where much of these municipalities present a weaker demographic structure in qualitative terms (more aged populations).

Along this paper we present a tool that we used to understand the different factors affecting the risk a given node presents to be infected. Here, we just tried to understand the first wave of the COVID-19 pandemic in Galicia (Spain) although we also present evidences of the accuracy of the method to explain the second wave. The developed tools can be potentiality used as a workbench to test different policies and their implications in the risk factors at a node level in order to prepare for future COVID-19 waves and new pandemics.

## Methods

### Galician spatial network

Without entering in verbose definitions, a complex network^53^ is a mathematical object represented by a graph, this is, a set of elements or *nodes* connected by links (also called *edges*). In the present case, the nodes are the different municipalities in the territory considered and the links establish the type of interactions between nodes. The interaction structure of the nodes within our particular complex network is given by the weighted adjacency matrix $M$, which we have constructed as a function of the council populations and the squared time distance between any two of them,

1 $$M_{ij} = \frac{{N_{i} N_{j} }}{{t_{ij}^{2} }} = w_{ij}$$

being $N_{i}$ and $N_{j}$ the population of any two locations $i$ and $j$, $t_{ij}$ the travel-time distance in car between them (that takes into account the real conditions of road infrastructures between nodes) and w_ij_ is the so-called weight of the edge connecting the nodes. With this kind of undirected symmetric adjacency matrix, we are able to build a heterogeneous network where each node has a unique set of weights or connectivity pattern. This will cause that each node experiences the dynamical process in a different way. The weights of the edges aim to model the flux of commuters from one node to another. In the absence of actual commuting census data, we considered more likely the exchange of travelers between heavily populated cities (the capital cities of the territories) and surrounding areas. According to these weights, we will speak about the node strength as the sum of all the weights of the connections of that node:

2 $$s_{i} = \mathop \sum \limits_{j}^{n} M_{ij} = \mathop \sum \limits_{j}^{n} w_{ij}$$

We have considered the time distance instead of the geometrical distance because of the Galician orography as well as the road infrastructures, that eventually might result in significantly larger travel times between neighboring locations than the expected from their relative physical distance^54^. In the case of the Atlantic coast, where the coast is segmented due to abundant estuaries, travel-times from one city to the other in opposed locations of the estuary takes longer by car than it would take on a boat (the straightforward direction). Of course, we have considered the time distance by car since it is a more likely option for travelling than the boat one. These time distances were estimated using the *Open Street Map API*^55^, and all geographical and demographical information about each node is extracted from the Galician Institute of Statistics^56^.

### Epidemiological model

To describe the infection and transmission process of the COVID-19 pandemic, many different models have been considered and yet there is no clear consensus on how many compartments should the model contain. Increasing or reducing the number of compartments is often determined by the final goal of the research. With our aim at hand, we model the epidemic spread across the subpopulations with the SAIRP deterministic model, a variation of the classic SEIR model that while maintaining certain simplicity was successfully applied in the case study of Portugal^38^. This model is composed of five differential equations that categorizes the human populations into five exclusive compartments, each governed by a set of transition rules. Basically, susceptible individuals *S* can become infected by interaction with either asymptotical *A* or symptomatic infected *I* individuals. This process occurs with a transmission rate $\beta$, for interactions with individuals of both categories. The asymptomatic ones become symptomatic infected with a rate $\nu$, while symptomatic become removed or recovered with rate $\mu$. The distinctive feature of this model compared to a typical SEIR is the category of protected individuals P. A fraction $p$ of susceptible individuals becomes protected with a transition rate $\phi$, but at each time step a fraction $m$ of the protected individuals becomes susceptible again with a transition rate $w$. We believe that this factor offers a suitable approach for modelling the increasing measures of non-pharmaceutical interventions used to control the propagation of the virus observed in the current times, such as social distancing, protective facial masks or mobility restrictions. The detailed equations are shown below.

### Coupling with the complex network

The above-described epidemiological model is now considered for each of the nodes in our complex network. Thus, the population of each municipality (node) is subdivided in all five compartments, with all individuals belonging initially to the susceptible compartment. Then, an initial seed of infected individuals is introduced randomly in the network and the disease propagates from there both locally and globally through the nodes of the network via the connections. Mathematically, this means that for each one of the nodes, there are a set of five nonlinear dynamical equations. The equations in all the nodes are coupled via an additional term that accounts for the network interaction. The network term only contributes positively to the transmission of the disease and represents both the interaction between susceptible residents with commuting infected individuals and vice versa. The larger the weight of the edge, the larger the amount of these interactions and the stronger the influence that highly infected nodes exert on their connections. A parameter $d$ was included in order to modulate the impact of these weights on the spreading dynamics. The model equations for any node $i$ are presented in (3).

3 $$\left\{ {\begin{array}{*{20}l} {\dot{S}_{i} = - \beta_{i} \left( {1 - p_{i} } \right)\left( {\frac{{A_{i} }}{{N_{i} }} + \frac{{I_{i} }}{{N_{i} }}} \right)S_{i} - \phi p_{i} S_{i} + wmP_{i} - d\beta S_{i} \mathop \sum \limits_{j = 1}^{n} M_{ij} \left( {\frac{{A_{j} + I_{j} }}{{N_{j} }}} \right)} \hfill \\ {\dot{A}_{i} = \beta_{i} \left( {1 - p_{i} } \right)\left( {\frac{{A_{i} }}{{N_{i} }} + \frac{{I_{i} }}{{N_{i} }}} \right)S_{i} - \nu A_{i} + d\beta S_{i} \mathop \sum \limits_{j = 1}^{n} M_{ij} \left( {\frac{{A_{j} + I_{j} }}{{N_{j} }}} \right)} \hfill \\ {\dot{I}_{i} = \nu A_{i} - \mu I_{i} } \hfill \\ {\dot{R}_{i} = \mu I_{i} } \hfill \\ {\dot{P}_{i} = \phi p_{i} S_{i} - wmP_{i} } \hfill \\ \end{array} } \right.$$

The meaning of the parameters is described in the previous subsection. So far, the only difference between nodes lies in their total population $N_{i}$ and their connectivity pattern given by the adjacency matrix $M$. Mobility is important but we also notice that nodes with the same number of individuals but different social and economic activities should behave differently. Just to name an example in the study case in Galicia, our model has to be able to differentiate a village on the countryside from one located on the shore with a harbor where there is usually more economic activity. In the following, we describe the different layers of information that we incorporated in the above model and that introduce the modulations on the $\beta$ and $p$ parameters for each node (hence, the subindex $i$ in the reaction terms of the equations). For the network term coupling the municipalities, we leave the global infectivity $\beta$ from the fitting, since using specific transmission rates among municipalities was not possible with the available data at the time.

### Layers of information incorporated into the epidemic model

Apart from the mobility data already incorporated into the model via the adjacency matrix, there are other layers of information that are important to consider as they may play a major role in the evolution of a pandemic.

Three airports are located in the studied region of Galicia in the most populated cities of A Coruña, Vigo and Santiago de Compostela. The net flux of passengers is obtained monthly from the national air-traffic statistics^57^. For simplicity, the flux was considered homogeneous in time and the flux of incoming travelers was considered to be half of each month’s total. This number of travelers is incorporated into the local node population with a distribution within each category as expected from the daily distribution of infected at the country level.

The region studied is mostly surrounded by the ocean and its economy strongly depends on ocean-related activities, thus, human interactions in the coast locations are also important. In order to incorporate this effect in the model by considering an effective infective transmission rate, $\beta$, that it is modulated following,

4 $$\beta_{i} = \beta + \beta_{port,i}$$

The additional term included, $\beta_{port,i}$, considers that the coastal cities are more prone to get infected due to interaction with the different ships arriving at the port. Here, it is important to distinguish the different types of economic activities in these coastal locations. Some of them have an intense economic activity while others are used mostly for leisure. In order to incorporate it into the model we considered the average age of each location, $y_{i}$, (extracted from demographic data) as an indicator of the level of economic activity in the node. Locations with a high value of the average age tend to be inhabited by retired senior citizens that use the port facilities mostly for leisure while younger populations are more involved in economic activities like fishing, trading, etc., that imply a higher risk of infection. All this is incorporated in the term,

5 $$\beta_{port,i} \left( {y_{i} } \right) = \left\{ {\begin{array}{*{20}l} {\beta ,} \hfill & {y_{i} < 18} \hfill \\ {\beta - \beta \frac{{\left( {y_{i} - 18} \right)}}{47},} \hfill & {18 > y_{i} > 65} \hfill \\ 0 , \hfill & {y_{i} > 65} \hfill \\ \end{array} } \right.$$

Note that we considered, as a first approximation that the infection rate linearly decreases with the average age of the location, with a saturation at the official retirement age (65 years). Of course, in the rest of localities the infective transmission rate remains $\beta_{i} = \beta$, this is, the one obtained from the regional fitting.

Next, we incorporated into the model information of the socioeconomic activity of each node, and we considered that those nodes that are more active have a higher rate of social mixing and interaction among individuals, situation that fosters the propagation of the virus. We can easily incorporate this rate of activity in the model by making the fraction of protected individuals $p$ a function dependent of the mean age $y_{i}$ at each node $i$,

6 $$p_{i} \left( {y_{i} } \right) = \left\{ {\begin{array}{*{20}l} {\frac{1}{65} y_{i} ,} \hfill & {y_{i} < 65} \hfill \\ {1,} \hfill & {y_{i} \ge 65} \hfill \\ \end{array} } \right.$$

Note that retired population (above 65 years old) usually takes extreme protective measurements as they are the more vulnerable and this is reflected in the model equation.

**Figure 8:**
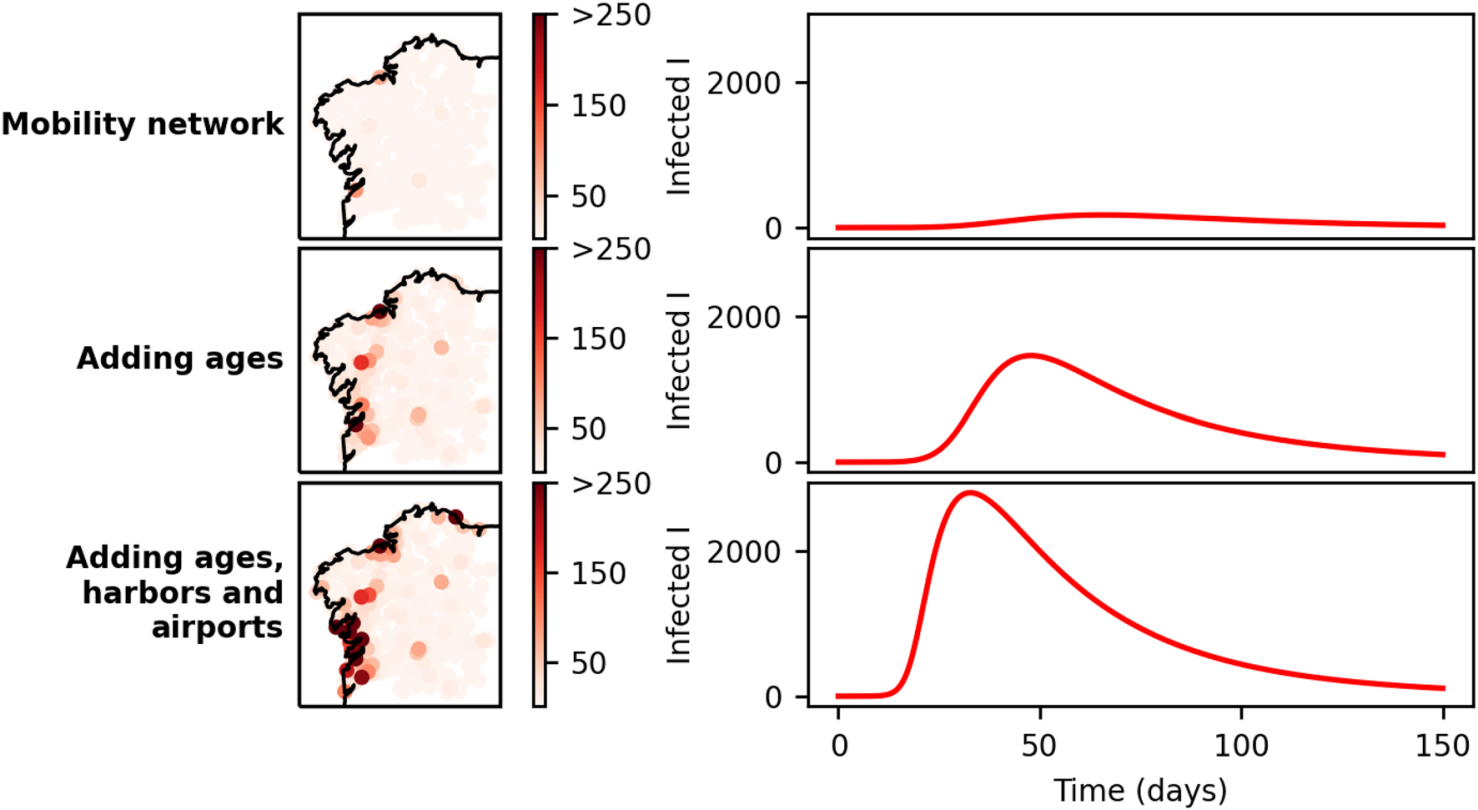
Different levels of complexity on the network model. The left panels show the normalized values of the infection peak for each one of the nodes, distributed spatially. The right panels show the time evolution of the infected individuals for one specific node, Burela, situated on the northeastern coast. To start the simulation, an initial infected seed was placed into each node.

The different layers of information considered here actually contribute unevenly to the infection curves. This can be observed in Fig. 8 where the local time evolution of the infected compartment is represented for a particular coastal node with the increasing layers of complexity from top to bottom. Left panels display the spatial distribution of the epidemic incidence. When the age or economic dynamicity is incorporated (central panel), the effect becomes more pronounced. Once the port activity is incorporated, the description of the epidemic in the coastal areas becomes more complete. Observing the panel on the bottom left, it can be grasped the emergence of two main hubs of infection, both on the extremes of the western coast and mainly influenced by the two most populated cities in the region, Vigo (south) and A Coruña (north). Since these cities conform the main hubs of economic and industrial activity it could be expected that their urban and suburban areas would suffer the worst outcome of the pandemic, and this is mainly what the network model captures. Eastern nodes that are also highly affected by the pandemic (shown in brighter red on the map) belong to the capital cities of those provinces, and to high-activity harbor nodes on the north-eastern coast.

Regarding the simulations, we devised the following scheme to obtain a statistical representation of those nodes that present the higher risk of an outbreak according to their particular conditions. First, with all the individuals placed on the susceptible compartment, we initiate the simulation with a seed of $n_{I}$ infected individuals distributed randomly (with uniform probability) among all the nodes. Then, we integrate Eq. (7) in time with a fourth order Runge–Kutta algorithm, until the dynamics of the infection have died out and all the individuals that were infected are placed on the removed compartment. As a measure of the impact of the disease on every node, we use the cumulative sum of symptomatic infected individuals. This quantity shows the impact of the infective transmission inside each node, giving us a tool to compare the evolution of the epidemic within the network. Finally, we repeat several times the simulation with different randomly-chosen initial seeds until we obtain enough statistical significance.

To assess the risk of each node to develop an outbreak, we proceed as follows. For each simulation, we sort the nodes as a function of this cumulative sum or total number of symptomatic infected individuals (from higher to lower) and keep for example, the first fifty nodes of each simulation (nodes with the larger number of symptomatic infected individuals). Following this example, if the number of simulations were to be 1000, we should have made a ranking of the nodes for each simulation and elaborate a table of 1000 rows and 50 columns. From this table, we count how many times each node appeared among the whole set of rankings, and we sort them again in terms of this counting. Those nodes with a higher value count will be those assigned with the highest risk. More precisely, we use this count as a measure or the *risk factor* by normalizing them to the number of carried simulations. If the risk factor equals 1, it means that the node appeared among the top infected nodes in all simulations, while a risk factor of 0 means that the node was never among them. Note that in this estimator the actual information on the size of the infection is discarded, since our aim is to assess if the municipality has a risk of being a hotspot rather than giving an accurate and quantitative prediction of the peak.

### Growth factors and linear stability analysis

In this section, we explain the linear stability approach to estimate some risk factors without numerically simulating the set of Eq. (3). First, we perform a simplification to the set of Eq. (3) considering that the total population of any node $N_{i}$ is constant over time, this is, $S_{i} \left( t \right) + A_{i} \left( t \right) + I_{i} \left( t \right) + R_{i} \left( t \right) + P_{i} \left( t \right) = N_{i}$ at any time $t$. Thus, the number of recovered individuals can be computed at any time $t > 0$ asThe SAIP simplified model is, then, described by the following set of equations:

$$R\left( t \right) = R\left( 0 \right) + \delta \mathop \smallint \limits_{0}^{t} I\left( u \right)du$$

7 $$\left\{ {\begin{array}{*{20}l} {\dot{S}_{i} = f\left( {S_{i} ,A_{i} ,I_{i} ,P_{i} } \right) - d\beta S_{i} \mathop \sum \limits_{j = 1}^{n} M_{ij} \left( {\frac{{A_{j} + I_{j} }}{{N_{j} }}} \right) } \hfill \\ {\dot{A}_{i} = g\left( {S_{i} ,A_{i} ,I_{i} ,P_{i} } \right) + d\beta S_{i} \mathop \sum \limits_{j = 1}^{n} M_{ij} \left( {\frac{{A_{j} + I_{j} }}{{N_{j} }}} \right)} \hfill \\ {\dot{I}_{i} = h\left( {S_{i} ,A_{i} ,I_{i} ,P_{i} } \right)} \hfill \\ {\dot{P}_{i} = z\left( {S_{i} ,A_{i} ,I_{i} ,P_{i} } \right)} \hfill \\ \end{array} } \right.$$

where $f, g, h$ and $z$ denote the nonlinear functions in Eq. (3). Let now us consider any of the possible steady states of the system and name it $\left( {\overline{{S_{i} }} ,\overline{{A_{i} }} ,\overline{{I_{i} }} ,\overline{{P_{i} }} } \right)$. If we introduce in each node a small perturbation to the node’s state

$$\left( {S_{i} ,A_{i} ,I_{i} ,P_{i} } \right) = \left( {\overline{{S_{i} }} ,\overline{{A_{i} }} ,\overline{{I_{i} }} ,\overline{{P_{i} }} } \right) + \left( {{\updelta }S_{i} ,\delta A_{i} ,\delta I_{i} ,\delta P_{i} } \right)$$

we can write the equations for the perturbations as,

8 $$\left\{ {\begin{array}{*{20}l} {\dot{\delta }S_{i} = \left( {f_{S} - d\beta \mathop \sum \limits_{j = 1}^{n} M_{ij} \left( {\frac{{\overline{{A_{j} }} + \overline{{I_{j} }} }}{{N_{j} }}} \right)} \right)\delta S_{i} + f_{A} \delta A + f_{I} \delta I_{i} + f_{P} \delta P - d\beta \overline{{S_{i} }} \mathop \sum \limits_{j = 1}^{n} M_{ij} \left( {\frac{{\delta A_{j} + \delta I_{j} }}{{N_{j} }}} \right)} \hfill \\ {\dot{\delta }A_{i} = \left( {g_{S} + d\beta \mathop \sum \limits_{j = 1}^{n} M_{ij} \left( {\frac{{\overline{{A_{j} }} + \overline{{I_{j} }} }}{{N_{j} }}} \right)} \right)\delta S_{i} + g_{A} \delta A_{i} + g_{I} \delta I_{i} + g_{P} \delta P + d\beta \overline{{S_{i} }} \mathop \sum \limits_{j = 1}^{n} M_{ij} \left( {\frac{{\delta A_{j} + \delta I_{j} }}{{N_{j} }}} \right)} \hfill \\ {\dot{\delta }I_{i} = h_{A} \delta A_{i} + h_{I} \delta I_{i} } \hfill \\ {\dot{\delta }P_{i} = z_{S} \delta S_{i} + z_{P} \delta P_{i} } \hfill \\ \end{array} } \right.$$

where subscripting represents the partial derivative over the variable (e.g., $f_{S} = \frac{\partial f}{{\partial S}}$). On the other hand, the perturbation of each one of the variables can be expanded over the set of eigenvectors of the Laplacian matrix of the network^39^, in close analogy to the classical case of continuous media where non-uniform perturbations can be decomposed into a set of spatial Fourier modes that represent plane waves with different wavenumbers^58^. In our case, since the Laplacian matrix is closely related to the adjacency matrix by the relation $L_{ij} = M_{ij} - \delta_{ij} k_{i}$, with $\delta_{ij}$ being the Kronecker delta and $k_{i}$ the connectivity of node $i$, we follow the same approach as in^39^ but with the adjacency matrix without any loss of generality. The eigenvalues ${\Lambda }_{\alpha }$ and eigenvectors $\Phi ^{\alpha } = \{ \phi_{1}^{\alpha } , \ldots ,\phi_{n}^{\alpha }$} of the adjacency matrix $M_{ij}$ for each mode $\alpha$ are given by $\mathop \sum \limits_{j = 1}^{n} M_{ij} \phi_{j}^{\alpha } = {\Lambda }_{\alpha } \phi_{i}$, with $\alpha = 1, \ldots ,N$. Expanding the perturbations $\delta X_{i}$ over the set of adjacency eigenvectors

9 $$\delta X_{i} = \mathop \sum \limits_{j = 1}^{n} c_{\alpha } \exp \left( {\lambda_{\alpha } t} \right)\phi_{i}^{\alpha }$$

the set of Eq. (8) is transformed into a set of $n$ independent equations for the different normal modes, resulting in the eigenvalue problem,

$$\lambda_{\alpha } \left( {\begin{array}{*{20}c} 1 \\ {B_{\alpha } } \\ {C_{\alpha } } \\ {D_{\alpha } } \\ \end{array} } \right) = \left( {\begin{array}{*{20}c} {f_{S} - d\beta \mathop \sum \limits_{j = 1}^{n} M_{ij} \left( {\frac{{\overline{{A_{j} }} + \overline{{I_{j} }} }}{{N_{j} }}} \right)} & {f_{A} - d\beta \overline{{S_{i} }} {\Lambda }_{\alpha } } & {f_{I} - d\beta \overline{{S_{i} }} {\Lambda }_{\alpha } } & {f_{P} } \\ {g_{S} + d\beta \mathop \sum \limits_{j = 1}^{n} M_{ij} \left( {\frac{{\overline{{A_{j} }} + \overline{{I_{j} }} }}{{N_{j} }}} \right)} & {g_{A} + d\beta \overline{{S_{i} }} {\Lambda }_{\alpha } } & {g_{I} + d\beta \overline{{S_{i} }} {\Lambda }_{\alpha } } & 0 \\ 0 & {h_{A} } & {h_{I} } & 0 \\ {z_{S} } & 0 & 0 & {z_{P} } \\ \end{array} } \right)\left( {\begin{array}{*{20}c} 1 \\ {B_{\alpha } } \\ {C_{\alpha } } \\ {D_{\alpha } } \\ \end{array} } \right)$$

The set of solutions $\lambda_{\alpha }$ for each mode $\alpha$ are called *growth factors* and from their definition on (9) it can be grasped that they give information on how fast any perturbation will drive the system towards or apart from the steady state. If we consider this state to be the one where all the population is in the susceptible compartment, it can be easily perturbed by introducing a small number of infected individuals. If for each node, there is at least one of the $\lambda_{\alpha }$ with a positive value, this means that the infective dynamics will develop in that node and that an outbreak of the pandemic will take place. On the other hand, a negative value implies that the perturbation will die out and the node would remain in its steady state. Furthermore, the value of the growth factor gives us a notion of how quickly an outbreak will evolve. Note that each node has a different set of values of the growth factor and the dynamics of that particular node will be controlled by the largest value of $\lambda_{\alpha }$ (in absolute value). Thus, we will record the maximum positive value of $\lambda_{\alpha }$ for each node and use as a reference of how likely is to become infected.
